# Climate change and plant reproduction: trends and drivers of mast seeding change

**DOI:** 10.1098/rstb.2020.0379

**Published:** 2021-12-06

**Authors:** Andrew Hacket-Pain, Michał Bogdziewicz

**Affiliations:** ^1^ Department of Geography and Planning, School of Environmental Sciences, University of Liverpool, Liverpool L69 7ZT, UK; ^2^ Department of Systematic Zoology, Faculty of Biology, Adam Mickiewicz University in Poznań, Ulica Uniwersytetu Poznańskiego 6, Poznań, 61‐614 Poland; ^3^ INRAE, LESSEM, University Grenoble Alpes, 2 rue de la Papeterie, BP 76, Saint‐Martin‐d'Hères, 38400 France

**Keywords:** masting, mass fruiting, seed production, spatial synchrony, plant recruitment, plant fecundity

## Abstract

Climate change is reshaping global vegetation through its impacts on plant mortality, but recruitment creates the next generation of plants and will determine the structure and composition of future communities. Recruitment depends on mean seed production, but also on the interannual variability and among-plant synchrony in seed production, the phenomenon known as mast seeding. Thus, predicting the long-term response of global vegetation dynamics to climate change requires understanding the response of masting to changing climate. Recently, data and methods have become available allowing the first assessments of long-term changes in masting. Reviewing the literature, we evaluate evidence for a fingerprint of climate change on mast seeding and discuss the drivers and impacts of these changes. We divide our discussion into the main characteristics of mast seeding: interannual variation, synchrony, temporal autocorrelation and mast frequency. Data indicate that masting patterns are changing but the direction of that change varies, likely reflecting the diversity of proximate factors underlying masting across taxa. Experiments to understand the proximate mechanisms underlying masting, in combination with the analysis of long-term datasets, will enable us to understand this observed variability in the response of masting. This will allow us to predict future shifts in masting patterns, and consequently ecosystem impacts of climate change via its impacts on masting.

This article is part of the theme issue ‘The ecology and evolution of synchronized seed production in plants’.

## 1. Introduction

The structure and composition of future vegetation depend not only on the impacts of climate change on plants' mortality but also on the processes determining recruitment, including seed production and establishment [1–3]. Recruitment is noisy over space and time, but recent research has indicated both increases and decreases in long-term average seed production [4–11]. In many plants, recruitment depends not only on mean seed production, but on the synchronous high interannual variability in seed production among individuals and populations, i.e. mast seeding [12]. In masting plants, recruitment occurs mainly after mast years, when seed predators are satiated and higher pollination efficiency during mass flowering increases seed viability [13–16]. Thus, the breakdown of masting can offset gains in recruitment that would otherwise be predicted by temporal increases in seed production [8,17] (box 1). Mast seeding is reported in species in boreal and temperate biomes of North America, Europe, Asia, South America and Oceania, and in tropical systems including tropical woodland, neotropic rainforests and in southeast Asia where masting species dominate lowland dipterocarp forests [18,19]. Masting is an important driver of forest regeneration dynamics across biomes. Therefore, predicting the long-term response of global vegetation dynamics to climate change requires understanding the response of masting to changing climate.

Box 1. **Changes in masting determine the fitness consequences of increased reproductive effort.**

Increased investment in reproduction does not necessarily translate into higher individual fitness or population-level reproductive success when it is accompanied by changes in masting, as demonstrated by Bogdziewicz *et al*. [8]. They showed that mean seed production in UK beech woodlands increased significantly over the period 1980–2018 in association with warming summer temperatures. However, the increase in seed production was accompanied by declining interannual variability and synchrony of seed production—a ‘breakdown’ in masting. The breakdown in masting relaxed suppression of the main seed predator of beech (*Cydia fagiglandana*) so that seed predation rates increased from approximately 1% in the 1980s to more than 40% in recent years. Likewise, the decline in flowering synchrony reduced pollination success by 34% over four decades. As a consequence of these changes in the economies of scale of masting, by the end of the study each tree was required to produce, on average, five flowers for each sound seed that reached the forest floor, while in the 1980s every second flower reached this stage. Over time the trees produced more seeds, but the benefits of increased investment in seed production were offset by the losses of reproductive efficiency associated with changes in masting.

Proximally, masting is triggered by species-specific weather cues such as temperature or precipitation deviations [20–23]. Seed production is also limited by climate-dependent resource availability [4,24]. Consequently, models predict that masting will be sensitive to climate change, but the direction of that change is uncertain [21,25–28]*.* This is unsurprising as climate change effects on seed production will result from the interaction of variable regional climate trends (e.g. local rate of warming, or change in moisture) and interspecific diversity in the proximate mechanisms that link weather and masting [29]. For example, high temperature promotes reproduction in *Fagus sylvatica* [30], but may block it in *Fagus crenata* [31]. Furthermore, internal resources limit masting, and populations with lower resource availability have generally higher interannual variability of seed production [18,32]. However, the limiting resource is likely to vary among populations, and we expect climate change to have spatially varying effects on these limiting resources. For example, in mesic habitats, global climate change may reduce interannual variation in seed production by increasing carbon availability, but increase variation where water is limiting. This predicted variability in masting responses to climate change is currently poorly understood. Furthermore, detecting trends in masting and attributing them to climate change is challenging owing to the lack of long-term data required to detect changes in highly variable time series. Furthermore, older, and larger plants can mast more frequently and show higher synchrony, further complicating efforts to isolate the effect of climate change [33,34,35]. Recently, data and methods have become available, allowing the first assessments of long-term changes in masting. We review these studies to search for evidence for a fingerprint of climate change on mast seeding, discuss the drivers and impacts, highlight challenges and suggest ways forward.

## 2. Fingerprints of climate change effects on mast seeding

Masting is quantified using a number of metrics that reflect different features of pulsed reproduction [36,37]. The features include interannual variation, temporal autocorrelation, synchrony among individuals and populations, average seed production and the frequency of mast years. These features of mast seeding—or masting ‘traits’—arise in response to selective pressures and economies of scale associated with concentrating reproduction into occasional pulses [19,32]. There is no *a priori* reason to expect that all masting traits covary, including in their response to climate change [37]. For example, individuals with decreasing interannual variation will not necessarily be those with declines in synchrony. Consequently, it is important to identify the most appropriate metric when quantifying masting change for any particular study system, including when considering the consequences of changes in masting for plant fitness and the wider ecosystem functioning. So far, the majority of studies examined temporal changes in the frequency of mast years and in mean seed production, often as a consequence of limited data. This is an important first step, but progress depends on systematic coverage of all aspects of mast seeding and the identification of plant traits, environments and geographies that may structure variation in masting response [38]. Individual plant data are valuable as they allow tracing of how changes in each of the masting patterns at the individual-level scale up to changing patterns at the population level.

In reviewing the evidence for climate change impacts on masting, we divide our discussion into the main characteristics of mast seeding: interannual variation, synchrony, temporal autocorrelation and mast frequency. We recognize that these characteristics are not ecologically or mathematically independent, and we discuss relevant examples below. Changes in average seed production have been discussed elsewhere and are not necessarily correlated to masting, so we do not discuss them here (see also box 1) [10,11]. In each section, we review the evidence for temporal change, discuss the role of climate change in driving it, identify the key consequences and discuss possible ways forward.

## 3. Interannual variation

High interannual variation in seed production is a defining characteristic of masting [39], and can be measured at the individual and population level. At the population level, interannual variation incorporates individual-level variation and within-population synchrony. From a plant fitness perspective, higher individual-level variation increases pollination efficiency and decreases seed predation, although this effect is greatest when combined with high population-level synchrony [40,41–43]. Interannual variation also results in resource pulses that drive the dynamics of both plant and animal populations and communities, such that mast seeding is among the most ubiquitous examples of terrestrial resource pulsing [44].

A global analysis of over one thousand time series belonging to 363 species showed an overall increase in population-level interannual variability over the past century [45]. Consistent with this global analysis, interannual variation in population-level seed production increased during the past half-century in six out of seven species studied in Poland, including: *Quercus petraea*, *Q. robur*, *Larix decidua*, *Picea abies*, *F. sylvatica* and *Abies alba*, but remained stable in *Pinus sylvestris* [34]*.* The trend in Poland was attributed to forest ageing more than to climate change [34]. By contrast, population-level interannual variation of seed production showed no change in North American conifers [38], and declined over the past four decades in *F. sylvatica* in England [8], in *Quercus crispula* in Japan [46] and in *Q. douglasii* in California [47]. The decline in population-level interannual variation in *F. sylvatica* was a consequence of decreases in both individual-level interannual variation and among-tree synchrony. The trends in *F. sylvatica* and *Q. crispula* correlated with warming, and are possibly driven by the less frequent veto of reproduction by weather events [48]. In *F. sylvatica*, individual trees appear to lose their responsiveness to weather cues as the cues become more frequent [49]. In *Q. crispula*, more frequent warm springs appear to facilitate efficient pollination, which likely leads to more regular reproduction [46].

The number of climate-sensitive mechanisms that regulate masting make the contrasting results unsurprising [50]. Nevertheless, we are aware of few attempts to understand this variability in response within a framework of theory-based hypotheses (although see [38], who test the prediction of the Δ*T* model). For example, the resource limitation hypothesis predicts that generally more stressful conditions are responsible for an increase in seed production variability [45,51]. Tests of the resource limitation hypothesis as an underlying driver of masting change can include comparing variability changes observed in resource-rich and resource-poor habitats. While some studies have used climate gradients to demonstrate that seed production variability is higher in more stressful environments [51], few studies have linked temporal changes in variability with temporal changes in climatic stress. Pearse *et al.* [45] showed no association between changes in variability and local rates of climate warming but did not account for differences in the effect of warming on stress. Future research may take advantage of altitudinal transects where warming might be expected to relax environmental stress at high elevations and increase stress at low elevations.

Alternatively, temporal trends in the variability of seed production might result from climate change-driven shifts in the frequency of reproductive vetoes, like droughts or frosts [52]. Accumulating theory allows characterization of specific vetoes to taxa and regions, like drought in oaks inhabiting dry lands and spring temperatures in oaks growing in mesic regions [22,28,53]. Comparing temporal trends in veto occurrence versus trends in seed production variability may prove illuminating.

Besides testing the drivers of masting change, it is important to understand how changes in interannual variability translate into recruitment and population growth of masting plants. For example, higher interannual variability leads to higher production of viable (pollinated and undamaged) seeds during mast events, but comes at costs of missed reproductive opportunities in low-seeding years [54]. This is particularly important when successful recruitment depends on the coincidence of masting and environmental conditions for seedling establishment [55]. Modelling studies indicate that less frequent masting (higher interannual variability) can alter successional pathways after disturbance, when the recruitment window for late-successional species is short [56]. Studies that estimate both sides of the trade-off are rare but crucial if we aim to understand the impact of changing variability on plant regeneration trajectories.

## 4. Synchrony

Synchrony of seed production operates at scales from local populations to continents [57,58]. Studies often recognize two scales: within- and among-site synchrony. Within-site synchrony is measured as the cross-correlation of seed production of individual plants within a study plot. This scale assesses coupling among neighbours that is relevant for pollination efficiency and the satiation of local seed predators [59]. Within-site synchrony results from shared individual responses to a synchronizing weather cue and via pollen-coupling [60]. Among-site synchrony is measured as cross-correlation of seed production among study plots, and ranges from regional to continental scales [58,61]. This scale is relevant for satiating mobile generalist seed predators [14] and has the potential to push and pull ecosystem dynamics at regional scales [62,63]. Theory suggests that regionally correlated weather variation (the Moran effect) is the main driver of synchronized seed production at this spatial scale [12,64].

We expect the climate to influence the spatial synchrony of masting via two mechanisms. First, climate change can disrupt the individual-level processes that generate within-population synchrony, which scales spatially via the Moran effect or pollen-coupling [65]. For example, warming may disrupt individual sensitivity to weather cues that regulate individual variability and synchrony [49]. Second, climate change may affect the spatial synchrony of climate at regional and continental scales [66]. The Moran effect then predicts an associated change in masting spatial synchrony, as has been observed with other ecological phenomena [67,68]. Unpacking temporal changes in reproductive synchrony thus requires the study of coupled fluctuations in both weather and seed production over geographical extents ranging from local field studies to continents. Despite the importance of synchrony for plant recruitment and community dynamics, and evidence that spatial synchrony is sensitive to temperature [64], temporal changes in spatial synchrony of seed production are poorly explored.

Among-site synchrony in seed production decreased during the past half-century in *Q. petraea*, *Q. robur*, *L. decidua* and *P. abies*, increased in *F. sylvatica*, and remained unchanged in *P. sylvestris* and *A. alba* [34]. The declines in oaks (*Quercus* sp.) were attributed to the declining spatial synchrony of spring weather. In that group, masting synchrony appears to be determined by a pollination Moran effect, i.e. pollination success is driven by variation in spring weather conditions [69,70]. Mechanisms responsible for changes in spatial synchrony of reproduction in *F. sylvatica* were less clear, as the weather cue that correlated with seed production showed no trends in spatial synchrony [34]. An increase in within-population synchrony of seed production was also reported in *Pinus pinea*, but the drivers were untested [71]. In other work, *F. sylvatica* populations in England showed a declining trend of within-population (among trees) and among-population synchrony of seed production over the past four decades [8]. In this system, synchrony break-down results from the disruption in the individual-level proximate process that generates within-population synchrony, i.e. weather cueing [49].

The synchrony of plant reproduction appears to be changing, both at local and regional scales. However, the role of changing climate in driving the trends remains to be resolved. The observed changes may be a response to changes in spatial synchrony of climate (Moran effect), or to changes in the underlying proximate mechanisms that create within-population synchrony and then scale to larger spatial scales. At regional scales, analysis of large-scale masting observational datasets using a geography of synchrony approach may illuminate the drivers of synchrony and its variability over time [72]. Where large-scale datasets based on observations of masting are not available, the use of cone-scars or dendrochronological methods may provide an opportunity to retrospectively assess changes in masting synchrony across scales [37,73].

Another challenge is to unpack the consequences of changing synchrony for recruitment and wider community dynamics. Declining synchrony has been demonstrated to decrease individual plant fitness as measured by viable seed production [40], but the next step is to link this with tree regeneration and population growth [74,75]. Trophic consequences of changes in synchrony are potentially substantial but remain unexplored. They include effects on animal migrations [63,76], the ability to produce regional risk forecasts of the spread of Lyme disease and hantavirus by rodents dependent on mast [77], and the planning of management and conservation actions in masting-dominated systems [78].

## 5. Temporal autocorrelation

Negative temporal autocorrelation measures the tendency of populations to alternate between years of high and low seed production, and is a common feature of seed production time series in masting species [36]. Temporal autocorrelation can be measured at all time lags. Zero autocorrelation at all time lags describes a time series with temporally random variability, while negative or positive autocorrelations imply a degree of cyclicity. The strength of autocorrelation does not, however, capture the magnitude of any variability. Masting studies have tended to focus on a time lag of 1 year (AR-1), where a strongly negative value is commonly used to infer the tendency for peaks in seed production to be followed by a years of low seed production. AR-1 can be interpreted as indirect evidence of resource depletion after mast years that limits seed production in years that follow [79]. In that context, it can be used to assess temporal changes in resource depletion [46]. From a fitness perspective, the specific sequence of low-seed and high-seed years should help escape predation [18], although the evidence for this is mixed [40,43].

Few studies have investigated the temporal change in autocorrelation and all those discussed here reported autocorrelation at lag 1 year (AR-1). In Poland, population-level temporal autocorrelation in seed production became more negative during the past half-century in *F. sylvatica*, *A. alba* and *P. abies*, and remained unchanged in *L. decidua*, *P. sylvestris*, *Q. petraea* and *Q. robur* [34]. In *Q. crispula* in Japan, temporal autocorrelation of seed production became less negative over the past four decades [46], while individual-level analysis reported no change in temporal autocorrelation in English populations of *F. sylvatica* [8]. Efforts to untangle the drivers of these changes in autocorrelation and test whether they are linked to climate change remain underdeveloped. For example, Shibata *et al*. [46] suggested a link between declining autocorrelation and rising temperatures and increased resource availability, but this has not yet been tested. Pesendorfer *et al.* [34] highlighted the relevance of changes in ontogeny, showing that the reported decline of autocorrelation in Polish *F. sylvatica* and *P. abies* forests was correlated with increased mean tree age.

Studies have indicated that the temporal autocorrelation of plant reproduction is changing, but interpreting these trends remains challenging. This is because our understanding of the causes of variation in AR-1 is still preliminary. Species with nutrient-poor vegetative tissues have more negative temporal autocorrelation compared to those with nutrient-rich tissues [32], but it remains unclear whether the observed variation in autocorrelation reflects altered resource dynamics. This will require new research to demonstrate a link between negative autocorrelation and resource depletion that limits reproduction in the following years. Additionally, more work is required to understand how changes in other masting metrics may correlate with changes in autocorrelation, including at time lags greater than −1 [19]. For example, changes in masting frequency (see §6) may change the lags at which autocorrelation is strongest, such that analysis of AR-1 provides only a partial picture of changes in autocorrelation and their implications for understanding the drivers of changes in masting (e.g. changes in resource dynamics).

## 6. Frequency

The frequency of masting (or the ‘return interval’ of mast years) refers to the average frequency of large seed crops, but it does not assume any regular periodicity to mast years. Consequently, the frequency of mast years is not necessarily related to autocorrelation. The importance of mast events for forest regeneration and as a food source of domestic animals meant it was probably the first masting pattern to be quantified and reported [80,81]. The concept was important in the development of evolutionary theories of masting [82]. However, while intuitive, quantifying the frequency of masting is problematic as it has traditionally required dividing continuous seed production data into the mast and non-mast years, while seed production follows a continuous rather than binomial distribution [83]. Nevertheless, as occasional large mast events are the key drivers of recruitment in many forest systems [13,84] and result in cascading effects on forest-based food-webs [85–87], changes in the frequency of mast years will have profound impacts on forest ecosystem dynamics (box 2).

Box 2. **Predicting the effect of changes on ecosystem dynamics**

Numerous studies have linked the pulses of resources associated with masting to wider cascading effects on communities, but few studies have explored the consequences of long-term changes in masting patterns for seed consumers. Using long-term monitoring data and a mechanistic model of oak masting, Touzot *et al.* [87] predicted an increased masting frequency in French oak forests over the next century. Models indicated that wild boar populations in these forests—under consistent hunting pressures—would remain stable under the current masting regime. However, because female breeding probability increased as a function of acorn availability, the predicted increase in masting frequency resulted in dramatic increases in predicted boar populations and their interannual fluctuations. While not explored in the study, such increases in boar populations would have dramatic cascading effects on forest food-webs, and on the regeneration of oaks and other species in these mixed forests.

**Figure 1.**
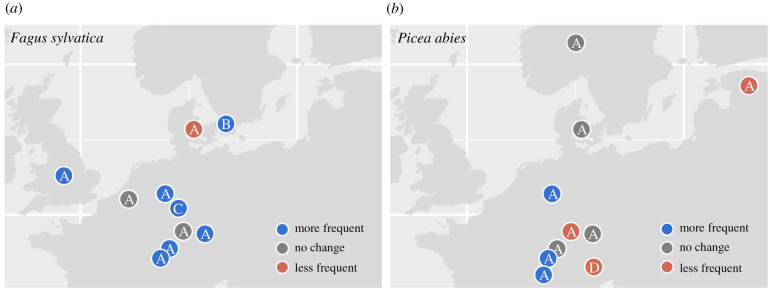
Reported changes in masting frequency across Europe. Most studies report increases mast frequency in *Fagus sylvatica* in recent decades (*a*), but no consistent response is reported for *Picea abies* (*b*)*.* A, Nussbaumer *et al*. [88]; B, Övergaard *et al*. [89]; C, Gruber [90]; D, Hacket-Pain *et al*. [91]. (Online version in colour.)

Several studies have reported an increase in mast year frequency in recent decades and have linked this correlatively with climate warming. European beech appears to be the best-studied species and the majority of evidence suggests that mast frequency has increased in recent decades (figure 1). The mast year interval during the period 1974–2006 was 2.5 years in Swedish beech forests, which appeared to be unprecedented compared to the previous three centuries, where the mast year interval was 4.1–6.0 years [89]. Comparing the late twentieth century with the early years of the twenty-first century, beech mast frequency increased in the UK, Germany and in Switzerland, but decreased in Denmark and did not change in Belgium [88,90]. In other species, the frequency of masting increased in *Q. crispula* in Japan [46], but no consistent shifts were found in *Q. robur* and *Q. petraea* in Europe [88]. In *P. abies* forests in northern Italy, the frequency of mast years, estimated at the population and individual level, declined in recent decades [91]. Four population-level mast years occurred during the first half of the study (1971–1992, average mast interval = 5.3 years), but only one mast year occurred in the second half of the study (1993–2012, mean interval = 21.0 years), with no mast years occurring since 1995. An analysis of a global network of 1086 time series for 363 species that found no global change in mast frequency over the past century [45], although this lack of a global signal may result from variation in the direction of change in frequency among species and populations.

It should be noted that detecting changes in masting frequency using short datasets is challenging. Multi-decadal mast records are rare, but analysis of European beech masting frequency based on regional aggregations of records [57] or tree-ring-based reconstruction [73] indicates substantial multi-decadal variability in mast frequency that is not clearly linked to long-term anthropogenic climate change.

Mast years represent pulses of reproduction and resources, thus understanding the climate change impact on mast frequency is crucial for predicting and managing ecosystem responses to climate change [92,93]. However, the direction of change is unlikely to be consistent. If the frequency of mast events is limited by resource availability, then climate change resulting in increased availability of limiting resources may increase the frequency of mast years [94]. However, evidence to support this assumption is mixed. Where temperature is limiting, high elevation populations do not consistently show less frequent mast years than their low elevation counterparts [6,95,96]. Across natural productivity gradients and in fertilization experiments, more favourable growing conditions are generally associated with larger seed crops in mast years rather than more frequent masting [24,89]. Climate manipulation experiments have not revealed a consistent response of masting frequency to reduced precipitation in drought-limited ecosystems [25,97]. On the other hand, a geographical transition from 2- to 3-year masting cycle in *Sorbus acuparia* appeared to follow the productivity gradient, with less frequent mast years where productivity was lower [94]. Similarly, higher nitrogen availability is associated with more frequent flowering in masting grasses [98]. Future work requires a framework of clear hypotheses for directional change in masting frequency, ideally across climate change gradients or experimental manipulations. Furthermore, such studies will benefit from methods that move beyond an event-based approach to assessing mast frequency, perhaps using wavelet analysis to identify time-varying periodicity in seed production time series [46,57].

## 7. Future directions

Several uncertainties should be prioritized in future research. Even in well-studied species, a coherent ‘fingerprint’ of climate change, akin to those detected in phenological or range-shifts studies [99,100], is difficult to detect. This is not surprising as changes in interannual variability, synchrony, temporal autocorrelation and masting frequency are expected to show diverse trends in response to climate warming, according to variation among species in the underlying mechanism regulating masting, and among populations according to the limiting factors of masting. However, such variations remain poorly understood. We have a developing understanding of how masting patterns vary among species and populations [19,32], and over climate gradients [51]. Nevertheless, whether the variation across climate space translates into variation over time as a result of climate change remains to be established. Pearse *et al*. [45] demonstrated an overall increase in interannual variation of reproduction across a global dataset representing 363 species, but a notable result was the large and unexplained variance in changes to interannual variation over recent decades. Thus, a priority for the next generation of studies based on increasingly extensive large-scale masting datasets will be to explain this variation, and identify species traits and regions that may structure this variation.

Metrics used to characterize masting are linked to individual fitness and population viability via the benefits gained through economies of scale, and to wider ecosystem dynamics via the characterization of resource pulses [37,43]. However, a full understanding of how masting responds to climate change is complicated as masting metrics are not independent. For example, a shift to more frequent mast years will reduce the interannual variability as measured by the coefficient of variation and will change the strength of autocorrelations at different time lags. Limited evidence so far indicates that spatial and temporal changes in masting patterns may not be correlated [34,37]. The next challenge is to understand if common responses exist and under what circumstances.

A major challenge is the attribution of observed masting changes to climate change. So far, studies are correlational rather than experimental, with causation to climate change inferred. For example, Pearse *et al.* [45] found no relationship between observed changes in interannual variability and local rates of climate warming across a dataset of 79 species, but this analysis was not able to control for the likely variation in response among species and habitats [11]. Analysis of the within-species masting response to local rates of climate change may prove a useful step forward, particularly where existing species-specific datasets cover gradients in the local rate of climate change. Nevertheless, masting responses will also depend on concomitant environmental changes including nitrogen deposition and CO_2_ fertilization, both of which may enhance forest productivity and relax nutrient limitation of masting [101]. The effect of large-scale climate oscillations on decadal trends in masting further complicates the attribution of changes in masting variability and spatial synchrony to anthropogenic climate change [55,57,102]. Untangling these interacting factors remains challenging. A small but growing number of studies have used experimental approaches in an attempt to isolate the effects of climate change on masting. In drought-limited ecosystems, long-term rainfall exclusion experiments indicate that increased drought stress does not result in strong effects on the interannual variability of seed or fruit production, even if mean seed production is reduced and the underlying mechanisms regulating reproduction are sensitive to reduced water availability [25,97,103]. Experimental studies manipulating climate in forest systems are logistically challenging, particularly over the time scales required to characterize masting. However, there are opportunities to leverage data collected in existing long-term warming or other manipulation experiments in forests, e.g. the SPRUCE (spruce and peatland responses under changing environments) experiment [104], and in systems that include masting shrubs or grasses. For example, data published from FACE (free air CO_2_ enrichment) experiments indicate that elevated CO_2_ increases mean seed production but does not change interannual variability [105]. Consequently, the still small number of experimental studies indicate that interannual variability of seed production may prove surprisingly robust to changes in CO_2_ or drought. Where the durations of climate manipulations are shorter they can still be used to investigate the response of proximate mechanisms of seed production to climate change [25], or better understand how shifts in resource allocation between reproduction and other plant functions will influence masting patterns [106–108].

A further challenge in attributing observed changes in masting to climate change is isolating the effects of climate change from those related to ontogeny [35]. Masting scales with plant size as larger plants reproduce more regularly, and therefore have less variable reproduction [33]. As the frequency of reproductive, failure years is related to synchrony, smaller plants also have lower synchrony with the rest of the population [33]. With increasing age, the masting patterns of individual plants will, therefore, shift independently of any exogenous drivers, with the same effect emerging at the population level if the distribution of plant size and age shifts over time. For example, the multi-decadal trends in reproductive variability, synchrony and autocorrelation in Polish forests broadly paralleled warming trends, but the main driver of the temporal evolution of masting in these forests was increasing forest age, resulting from the long-term impact of changes in forest management [34]. The challenge of isolating climate change and ontogenic effects is further complicated by their likely interaction. For example, climate change effects on fecundity in North American forests are dominated by the indirect effects of climate change on tree size [11]. While largely unexplored for masting, similar effects might be expected if climate change results in shifts in plant size distributions, particularly as most masting datasets used to assess reproduction-level reproduction are based on repeated measurements of marked individual plants, which increase in age through the monitoring period.

## 8. Conclusion

Predicting changes in mast seeding in response to climate change is a complex endeavour. It is not a ‘simple’ physiological process where trade-offs are balanced to maximize individual fitness by maximizing the rate of growth or the production of seeds, or minimizing the risk of mortality by balancing the investment of resources in growth, reproduction or defence. Instead, masting is a dynamic strategy that maximizes fitness based on varying allocation to reproduction [109]. In masting plants, strongly varying and synchronized reproduction has evolved to maximize pollination efficiency and reduce seed predation [40]. Climate change may result in changes to whole-plant resource availability and to the relative allocation of those resources to reproduction and other resource sinks [106,107], but neither of these processes will automatically result in changes in masting patterns—with the exception of mean reproduction.

To understand the response of interannual variability, synchrony, temporal autocorrelation and mast frequency to climate change, we must use a dual approach that combines the analysis of long-term monitoring datasets and targeted experimental studies. Multi-decade masting datasets are increasingly available. They now include high species replication and time series collected from sites distributed over large climate gradients, including across regions that have experienced varying rates of recent climate change [45,110]. Testing for changes in masting patterns in such datasets, combined with improved methods of climate change attribution, will enable characterizing masting responses to recent climate change. Such studies will enable a general understanding of likely responses of masting to climate change, including testing alternative hypotheses for masting sensitivity to climate change [21,27,38]. Nevertheless, predictions of future responses will require greater understanding of the finely tuned proximate mechanisms that generate these patterns at the individual and population levels [12,29,111]. In particular, we need to establish how these mechanisms respond to different aspects of climate change, including warming, drying, changes in interannual climate variability and the frequency of extremes, and other aspects of environmental change including atmospheric CO_2_ and nitrogen fertilization.

## Data accessibility

This article has no additional data.
